# A gauge of coral physiology: re-examining temporal changes in *Endozoicomonas* abundance correlated with natural coral bleaching

**DOI:** 10.1093/ismeco/ycae001

**Published:** 2024-01-12

**Authors:** Po-Shun Chuang, Sheng-Ping Yu, Po-Yu Liu, Ming-Tsung Hsu, Yu-Jing Chiou, Chih-Ying Lu, Sen-Lin Tang

**Affiliations:** Biodiversity Research Center, Academia Sinica, Taipei 115, Taiwan; Biodiversity Research Center, Academia Sinica, Taipei 115, Taiwan; School of Medicine, College of Medicine, National Sun Yat-sen University, Kaohsiung 804, Taiwan; Biodiversity Research Center, Academia Sinica, Taipei 115, Taiwan; Biodiversity Research Center, Academia Sinica, Taipei 115, Taiwan; Centre for Marine Science and Innovation, School of Biological Earth and Environmental Sciences, University of New South Wales, Sydney 2052, Australia; Biodiversity Research Center, Academia Sinica, Taipei 115, Taiwan; Molecular and Biological Agricultural Sciences Program, Taiwan International Graduate Program, National Chung Hsing University and Academia Sinica, Taipei 115, Taiwan; Graduate Institute of Biotechnology, National Chung Hsing University, Taichung 402, Taiwan; Biodiversity Research Center, Academia Sinica, Taipei 115, Taiwan

**Keywords:** *Endozoicomonas*, coral health, symbiont switching, coral microbiome, thermal bleaching, coral recovery

## Abstract

Bacteria contribute to many physiological functions of coral holobionts, including responses to bleaching. The bacterial genus, *Endozoicomonas*, dominates the microbial flora of many coral species and its abundance appears to be correlated with coral bleaching. However, evidences for decoupling of bleaching and *Endozoicomonas* abundance changes have also been reported. In 2020, a severe bleaching event was recorded at reefs in Taiwan, providing a unique opportunity to re-examine bleaching-*Endozoicomonas* association using multiple stony corals in natural environments. In this study, we monitored tissue color and microbiome changes in three coral species (*Montipora* sp., *Porites* sp., and *Stylophora pistillata*) in Kenting National Park, following the bleaching event. All tagged *Montipora* sp. and *Porites* sp. recovered from bleaching within 1 year, while high mortality occurred in *S. pistillata*. Microbiome analysis found no correlation of *Endozoicomonas* relative abundance and bleaching severity during the sampling period, but found a stronger correlation when the month in which bleaching occurred was excluded. Moreover, *Endozoicomonas* abundance increased during recovery months in *Montipora* sp. and *Porites* sp., whereas in *S. pistillata* it was nearly depleted. These results suggest that *Endozoicomonas* abundance may represent a gauge of coral health and reflect recovery of some corals from stress. Interestingly, even though different *Endozoicomonas* strains predominated in the three corals, these *Endozoicomonas* strains were also shared among coral taxa. Meanwhile, several *Endozoicomonas* strains showed secondary emergence during coral recovery, suggesting possible symbiont switching in *Endozoicomonas.* These findings indicate that it may be possible to introduce *Endozoicomonas* to non-native coral hosts as a coral probiotic.

## Introduction

Ocean warming due to climate change has raised great concerns about its impact on coral reefs globally. Bleaching refers to disruption of symbiosis between corals and photosynthetic dinoflagellates of the family Symbiodiniaceae, a phenomenon commonly observed in thermally stressed corals [1]. Since its first documentation in the 1980s, coral bleaching has been reported with increasing frequency, with three pan-tropical coral bleaching events in 1998, 2010, and 2016 [2-6]. Regional bleaching episodes have also been recorded from tropical reefs in Australia [7-10] and the Caribbean [11, 12] to subtropical regions such as Okinawa, Japan [13-15], and Taiwan [5, 16-18]. As photosynthates from dinoflagellate symbionts constitute the major carbon source in stony corals [19, 20], severe bleaching can devastate coral physiology. Subsequent coral mortality can also change the structure of coral reef ecosystems, impacting all associated species.

In addition to dinoflagellates, corals are associated with a great diversity of bacteria—collectively termed the coral microbiome. Bacteria are thought to participate in many physiological functions of coral holobionts, including ontogeny [21, 22], metabolism [23, 24], immunity [25], and stress tolerance [23, 26]. In both laboratory and field studies, coral bleaching has been associated with decreases in *Gammaproteobacteria* [27-29]. Increases in *Vibrio* bacteria have also been documented in several stony corals during bleaching [1, 27, 30-32]. In fact, early studies demonstrated that *Vibrio shiloi* and *Vibrio coralliilyticus* induce bleaching in *Oculina patagonia* and *Pocillopora damicornis*, respectively [33, 34]. However, our knowledge of functional associations between the coral microbiome and bleaching, especially for nonpathogenic symbionts, is still limited.

*Endozoicomonas* (*Gammaproteobacteria*; *Oceanospirillales*; and *Endozoicomonadaceae*) constitutes a dominant bacterial taxon in several corals [35-38]. Based on genomic evidence, recent studies have proposed that *Endozoicomonas* regulates various biological functions in corals, such as metabolism, signaling, and nutrient cycling, suggesting that these bacteria are potentially beneficial for corals [39, 40]. Using denaturing gradient gel electrophoresis and cloning techniques, Bourne *et al*. [30] first identified a correlation between *Endozoicomonas* abundance (identified as *Spongiobacter* sp. in that study) and zooxanthella density in *Acropora millepora.* Occurrence of microbiome changes prior to visible signs of bleaching led to the hypothesis that *Endozoicomonas* is an early indicator of coral health/stress [30]. Thereafter, several studies reported that *Endozoicomonas* abundance decreases during natural [28, 29, 41] or experimental bleaching in other corals [26, 27, 42]. However, decoupling of coral bleaching and *Endozoicomonas* abundance changes has also been observed. For instance, Núñez-Pons *et al*. [38] monitored microbiome changes in three stony corals (*Montipora capitata*, *Porites compressa*, and *Pocillopora acuta*) in Hawaii following a natural bleaching event in 2016 and found no significant correlation between *Endozoicomonas* relative abundance and bleaching severity in those corals*.* In laboratory experiments, dynamics of *Endozoicomonas* abundance in *Pocillopora verrucosa* and *Euphyllia glabrescens* were also independent of the bleaching induced by excess dissolved organic carbon (DOC) and dark treatments, respectively [43, 44]. These contradictory findings suggest that the association between bleaching and decreased *Endozoicomonas* abundance is probably more complicated and may depend on specific combinations of *Endozoicomonas* bacteria and host corals.

Interestingly, in *A. millepora*, Bourne *et al*. [30] also found high microbiome similarity between unbleached and recovered (post-bleaching) corals, suggesting that *Endozoicomonas* recovery accompanies coral recovery. As *Endozoicomonas* is rare in seawater [45] and shows strong host-specificity in symbiosis with corals [35], it may be that the taxonomic composition of *Endozoicomonas* remains relatively stable during bleaching recovery. Supporting this hypothesis, Pootakham *et al*. [28] showed clear evidence that, in *Porites lutea*, dominant *Endozoicomonas* bacteria returned to pre-bleaching densities during coral recovery without changing their relative dominance. However, as similar studies on other corals are still limited, whether the same phenomenon applies to other stony corals remains unknown. In 2020, an extremely warm summer caused intense coral bleaching in coral reefs around Taiwan, with 57%–84% of shallow (3 m depth) corals in Kenting, southern Taiwan, showing partial or complete bleaching [46]. Given that Kenting National Park is a marine protected area, this bleaching event provided an opportunity to study bleaching-*Endozoicomonas* correlation in multiple coral species with minimal anthropogenic disturbance. By monitoring tagged corals for 1 year following the bleaching event, we examined two hypotheses: (i) *Endozoicomonas* abundance in corals negatively correlates with bleaching severity; (ii) the taxonomic composition of *Endozoicomonas* bacteria does not change after natural bleaching.

## Materials and methods

### Coral and seawater sampling

In 2020 and 2021, we collected corals six times from reefs located in Kenting National Park in southern Taiwan. Sampling covered the bleaching event in August 2020 (herein, the bleaching month) and five times during the following year: September 2020, October 2020, November 2020, April 2021, and August 2021 (recovery months) to examine coral recovery. Fifteen bleached colonies of *Montipora* sp. (*N* = 5), *Porites* sp. (*N* = 5), and *Stylophora pistillata* (*N* = 5) were tagged in August 2020 and were monitored for tissue color changes in subsequent fieldwork. Colonies of the same species were selected at distances >5 m from one another to maximize genetic randomness. Photos of tagged colonies were taken to estimate bleaching extents based on categories defined in Fisch *et al*. [47]. At each sampling time, three fragments (2–3 cm^2^) from each tagged, living colony and 1 liter of seawater were collected, comprising a total of 243 samples (237 coral tissues + 6 seawater samples). Immediately after sampling, coral tissues were washed once with 0.22-μm-filtered natural seawater and were preserved in 99% ethanol. Coral tissue and seawater samples were transported at 4°C and were then stored at −20°C until DNA extraction.

### Deoxyribonucleic acid extraction and 16S amplicon sequencing

Genomic DNA was extracted from coral tissues and seawater samples (filtered through 0.22-μm membranes) using a modified cetyltrimethylammonium bromide method [48]. To construct 16S amplicon libraries, we first performed polymerase chain reaction (PCR) to amplify the V6-V8 hypervariable region of 16S rRNA gene using the 968F (5'-AACGCGAAGAACCTTAC-3′) and 1391R (5'-ACGGGCGGTGWGTRC-3′) primers with following PCR conditions: initial step at 94°C for 5 min, followed by 30 cycles of 94°C for 30 s, 52°C for 20 s, and 72°C for 45 s, and a final step at 72°C for 10 min. PCR products were tagged using DNA-tagging PCR, following the protocol in Chen *et al*. [49]. The 16S amplicon libraries that were constructed were submitted to Yourgene Health Co., Ltd (New Taipei City, Taiwan) for sequencing using a Miseq reagent kit v3 (300-bp paired-end sequencing; 600 cycles) on an Illumina MiSeq system.

### Amplicon sequence analysis

Quantitative Insights Into Microbial Ecology 2 (QIIME2) was used to analyze the 16S rRNA amplicon sequences [50]. Briefly, raw reads from Illumina MiSeq sequencing were first reoriented, primers were trimmed, and sequences were demultiplexed using the cutadapt plugin [51]. Demultiplexed reads were then truncated to 235 bp from both ends and were denoised using the DADA2 plugin [52]. To refine the sparseness of the amplicon sequence variant (ASV) abundance table, ASVs acquired from QIIME2 were reclustered to *k*-mer taxonomic units (KTUs) using the “ktusp” function of the KTU algorithm in the R environment (v4.2.1). This procedure has been proposed to improve biological explanations of microbiome data [53]. Taxonomy of each KTU was assigned based on the SILVA 138 SSU reference database using the “kaxonomy” function (annotation parameter: consensus = 0.5) in the KTU algorithm. KTUs affiliated with chloroplasts or mitochondria were removed, as were those affiliated with unclassified kingdoms or phyla. Libraries with <1000 remaining sequences (seven libraries) were removed from subsequent analyses.

### Statistical and biodiversity analyses of coral microbial communities

To examine microbiome structure, we first rarefied coral tissue and seawater libraries to 1000 sequences/library. Coral tissue libraries of the same colony at the same sampling time were then pooled and re-rarefied to 1000 sequences to remove pseudo-replicates, yielding a total of 85 merged libraries for subsequent analyses (30 for *Montipora* sp., 30 for *Porites* sp., 19 for *S. pistillata*, and 6 for seawater). However, as library merging tends to inflate estimates of species richness (Supplementary Table S1), pseudo-replicate removal in alpha diversity was conducted by calculating alpha diversity on a per-sample basis and by averaging over coral colonies for each sampling time. Alpha diversity was estimated with the Chao1 and Shannon indexes. Statistical analyses for group comparisons of alpha diversity were conducted using the Kruskal–Wallis test with Dunn’s *post hoc* test. Beta diversity of microbial communities was assessed using Bray–Curtis dissimilarity and was visualized with principal coordinate analysis (PCoA). Heterogeneity among communities was analyzed using Analysis Of Similarities (ANOSIM; permutations = 1000) in Mothur [54]. To examine *Endozoicomonas* abundance changes and composition shifts, low-abundance *Endozoicomonas* KTUs (<20 sequences across merged libraries) were removed to minimize stochastic errors. This resulted in 19 *Endozoicomonas* KTUs. Abundance changes of *Endozoicomonas* during the sampling period were analyzed using the Kruskal–Wallis test and Dunn’s *post hoc* test for coral species. Correlations between *Endozoicomonas* relative abundance and bleaching extent for each coral colony was examined using Pearson’s correlation test. A significance level of *α* = 0.05 was set for all analyses in this study. Given that sample numbers in this study were not high (*N* = 3 or 5 for each group), no *p*-value adjustment was conducted for multiple comparisons.

### Species identification and phylogenetic analysis of *Endozoicomonas k*-mer taxonomic units

Species-level taxonomy of 19 non-rare *Endozoicomonas* KTUs were identified by Blast search against the National Center for Biotechnology Information (NCBI) r/RNA/ITS database. A phylogenetic tree was reconstructed using the Maximum Likelihood method and the Tamura-Nei model based on representative sequences of these *Endozoicomonas* KTUs with 500 bootstrap replicates in MEGA11 [55]. 16S rRNA genes from nine *Endozoicomonas* bacteria (trimmed to the V6–V8 region) were included in the phylogenetic analysis as references, with an *Aliivibrio fischeri* 16S rRNA gene sequence (NR_029255.1) as the outgroup taxon.

## Results

### Coral physiology

**Figure 1:**
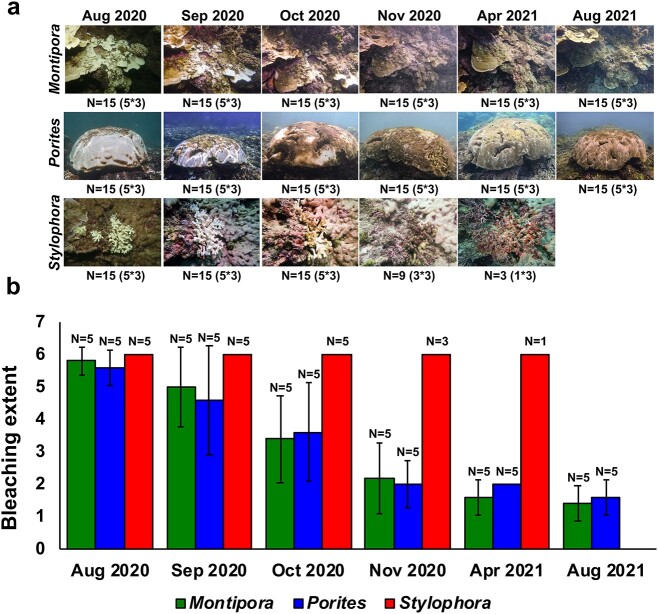
**Morphological changes of corals following the 2020 bleaching event**. (A) Representative photos of three coral species during the sampling period. Numbers of collected samples at each sampling time are indicated under photos, with numbers in parentheses indicating numbers of colonies * numbers of fragments. (B) Bleaching extents of three coral species during the sampling period. Data are presented as means ± standard deviations, and numbers of libraries at each sampling time are indicated. Coral species are labeled by genus.

In August 2020, intense coral bleaching was observed at Kenting National Park in southern Taiwan. Bleached colonies of *Montipora* sp., *Porites* sp., and *S. pistillata* were tagged and subsequently monitored for 1 year. Both *Montipora* sp. and *Porites* sp. showed signs of color recovery commencing in October 2020, and all tagged *Montipora* sp. and *Porites* sp. colonies visually recovered from bleaching in 2021 (mean bleaching extent: 1.65; Fig. 1). In contrast, *S. pistillata* showed no signs of color recovery following thermal bleaching. Mortality of *S. pistillata* was detected in November 2020, and all tagged *S. pistillata* colonies were dead and covered with algae in August 2021 (Fig. 1).

### Sequencing overview and bacterial composition

**Figure 2:**
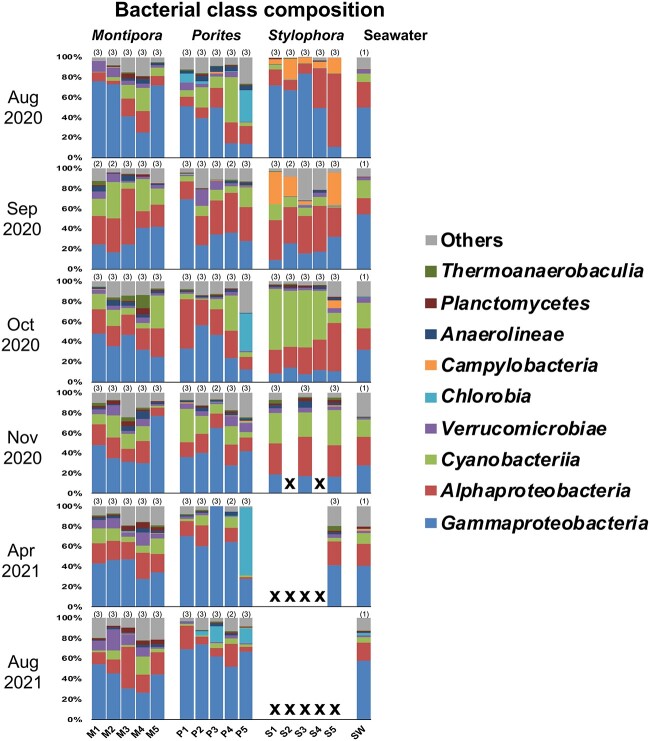
**Bacterial composition at the class level**. Data are presented for each coral colony/seawater at each sampling time, with numbers of collected samples indicated in parentheses. Bacterial classes with <1% relative abundances across all merged libraries are presented as “others”. M: *Montipora* sp.; P: *Porites* sp.; S: *S. pistillata*; SW: seawater.

Due to deaths of *S. pistillata*, only three colonies of *S. pistillata* were sampled in November 2020 for microbiome analysis and one colony in April 2021, while no *S. pistillata* colonies were sampled in August 2021. After data processing, seven libraries were removed from subsequent analyses due to low sequencing depth (<1000 bacterial sequences/library). This yielded a total of 2 280 673 bacterial sequences from 236 coral tissue and seawater libraries (1052–40 839 sequences/library), which were clustered into 10 044 KTUs affiliated with 115 bacterial classes. After data rarefication (1000 sequences/library) and pseudo-replicate removal, 5873 KTUs remained across 85 merged libraries. In both seawater and merged coral tissue libraries, *Gammaproteobacteria*, *Alphaproteobacteria*, and *Cyanobacteriia* were the dominant bacterial classes (Fig. 2). The merged dataset is available in Supplementary Table S2.

### Alpha diversity

**Figure 3:**
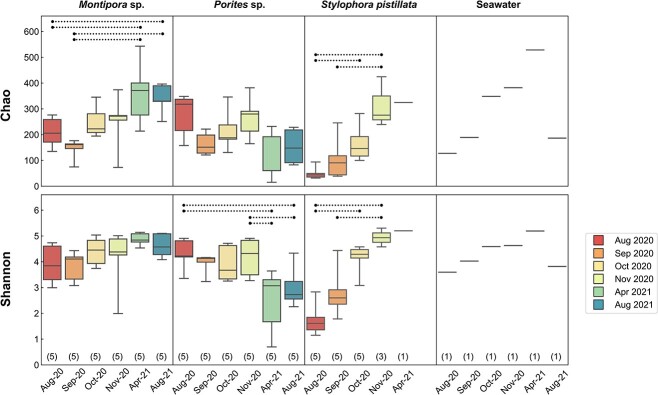
**Alpha diversity of bacterial communities in coral tissue and seawater libraries**. Chao1 and Shannon indexes were used to estimate alpha diversity. Boxes and whiskers indicate quartiles and full data ranges, respectively. Numbers of libraries at each sampling time are indicated in parentheses. Dashed lines indicate statistically significant differences (Dunn’s *post hoc* test; *p* < 0 .05).

During the sampling period, different patterns of alpha diversity changes were found among sample types (Fig. 3). In *Montipora* sp., both bacterial species richness (Chao1 index) and evenness (Shannon index) showed weak trends of increase during the sampling period, with significant differences identified in the Chao1 index for samples collected in August and September 2020 compared to those collected in 2021 (Supplementary Table S3). In contrast, *Porites* sp. showed decreasing trends in Chao1 and Shannon indexes during the sampling period. Significant differences were found in the Shannon index for samples collected in August and November 2020 compared to those in 2021. In *S. pistillata*, both species richness and evenness showed pronounced increases during the sampling period, which became significant in October 2020, compared to the bleaching month.

### Beta diversity

**Figure 4:**
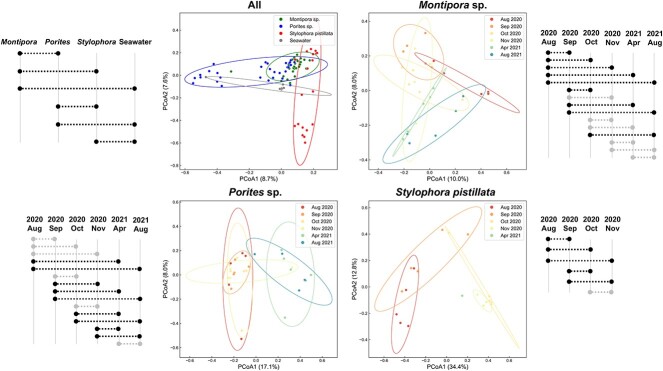
**PCoA plots of bacterial composition at the KTU level**. Pairwise comparisons are indicated with dashed lines on the side of each PCoA plot with statistically significant differences highlighted (ANOSIM; *p* < 0 .05).

Analysis of beta diversity showed significant differences among coral species and seawater (Fig. 4; ANOSIM; 1000 permutations; *p* < 0.05; Supplementary Table S4). In all three coral species, significant changes were identified during the study. In *Montipora* sp. and *S. pistillata*, multiple comparisons yielded significant differences in combinations both within and across years (for *Montipora* sp.), whereas in *Porites* sp., significant differences were found mostly in cross-year comparisons.

### *Endozoicomonas* composition

**Figure 5:**
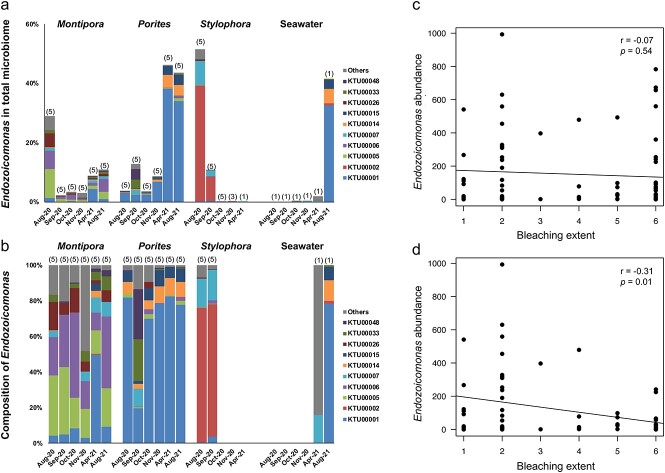
***Endozoicomonas*** **bacterial composition and correlation with bleaching extent**. (A) Percentages of *Endozoicomonas* bacteria versus total microbiome. (B) Percentages of *Endozoicomonas* bacteria versus total *Endozoicomonas* communities; Data are presented as averages for each sampling time, with numbers of libraries indicated in parentheses. KTUs with <20 sequences across merged libraries are not included and KTUs with <1% of the total *Endozoicomonas* community across merged libraries are presented as “others”. (C) Correlation of *Endozoicomonas* relative abundance and bleaching extent during the entire sampling period. (D) Correlation of *Endozoicomonas* relative abundance and bleaching extent with the bleaching month removed. Statistical significance of data correlation was examined using Pearson’s correlation test.

**Figure 6:**
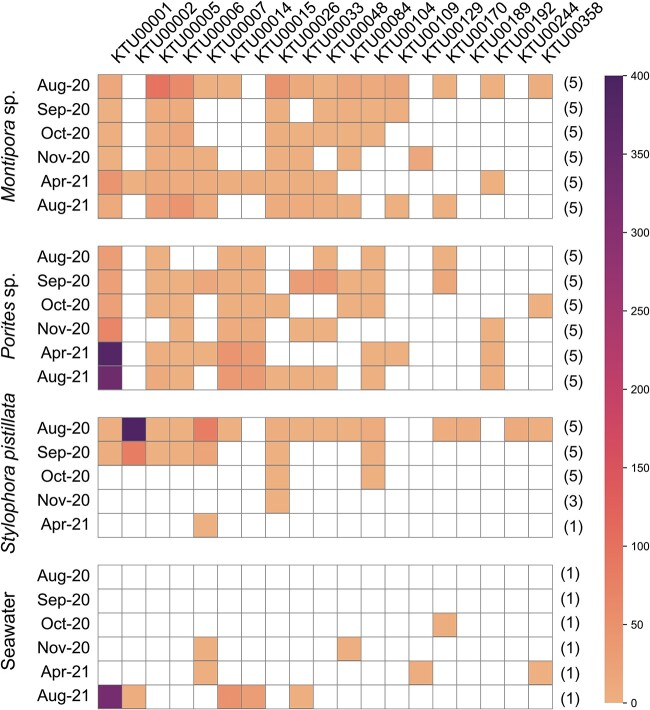
**Heatmap of** ***Endozoicomonas*** **KTUs in coral tissue and seawater libraries**. Data are presented as averaged relative abundances of *Endozoicomonas* KTUs for each sampling time, with numbers of libraries indicated in parentheses. Data of zero abundance are highlighted in white. KTUs with abundances <20 sequences across merged libraries were not included to avoid bias.

**Figure 7:**
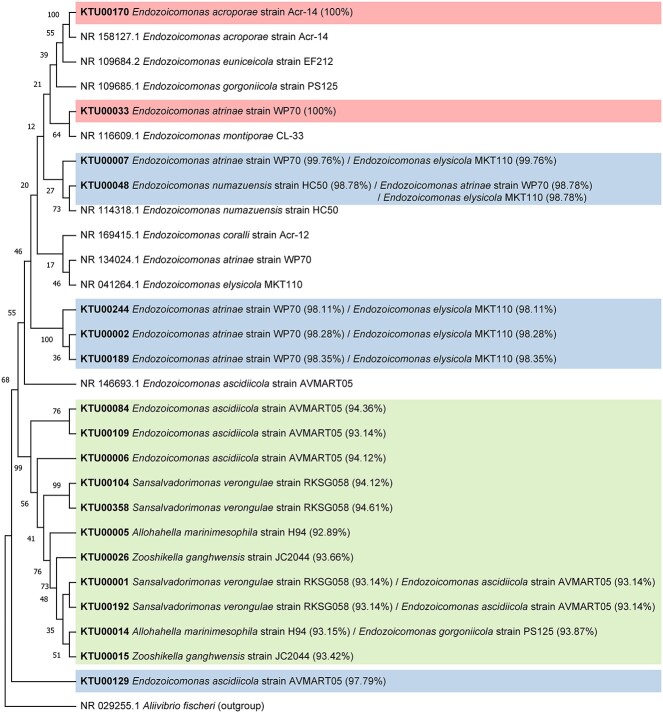
**Phylogenetic tree of** ***Endozoicomonas*** **KTUs in this study**. Sequences of publicly available *Endozoicomonas* bacteria were included as references, with *A. fischeri* (NR_029255.1) serving as an outgroup taxon. Best matches against the NCBI database are provided following KTU numbers with sequence identities in parentheses. Bootstrap values are presented at the node of each branch.KTUs with 100%, >97%, and <95% sequences identities to their best matches in the NCBI database are differently highlighted.

Nineteen KTUs were retained after removal of low-abundance *Endozoicomonas* KTUs. Total *Endozoicomonas* abundance varied significantly among sampling times for *Porites* sp. and *S. pistillata* (Kruskal–Wallis test; *p* < 0 .05; Supplementary Table S5). For *Montipora* sp. and *S. pistillata*, decreasing *Endozoicomonas* relative abundance occurred during the bleaching month and the first few recovery months. For *Porites* sp., the bleaching month showed the lowest *Endozoicomonas* relative abundance during this study. During recovery months, both *Montipora* sp. and *Porites* sp. showed increases in *Endozoicomonas* relative abundance, whereas *Endozoicomonas* became almost undetectable in *S. pistillata* after October 2020. Taxonomically, dominant *Endozoicomonas* KTUs varied between coral species (Fig. 5). During the sampling period, taxonomic composition of dominant *Endozoicomonas* remained relatively stable in the three coral species, with structural fluctuations occurring only sporadically (Fig. 5B). When comparing *Endozoicomonas* relative abundances and bleaching extents in the three coral species, no significant correlation was found during the entire sampling period (Fig. 5C). However, a significant, negative correlation was found when the bleaching month was excluded from the analysis (Fig. 5D). Disregarding abundances, several KTUs were present in corals in recovery months but not in the bleaching month (*Montipora* sp.: three KTUs; *Porites* sp.: eight KTUs; *S. pistillata*: zero KTUs; Fig. 6). A phylogenetic analysis and Blast search against the NCBI database showed that most *Endozoicomonas* KTUs in our data could not be clearly affiliated with known *Endozoicomonas* bacteria, with 11 *Endozoicomonas* KTUs showing <95% sequence identity to their corresponding best match sequences in the NCBI database (Fig. 7).

## Discussion

### Tissue color recovery after bleaching

In this study, we monitored tagged colonies of *Montipora* sp., *Porites* sp., and *S. pistillata* at Kenting National Park in southern Taiwan following an intense bleaching event in 2020. Bleaching is a common coral response to thermal stress, but it is also considered a means to rapidly adapt to changing environments [56]. According to this concept, bleaching averts intracellular accumulation of ROS generated by Symbiodiniaceae at elevated temperatures, allowing a coral to survive thermal stressors [57, 58]. When stresses abate, corals can re-establish the coral–Symbiodiniaceae symbiosis by either repopulating remnant photosynthetic dinoflagellates within their tissues or by capturing planktonic symbionts from ambient seawater [56, 59]. Commensurate with this notion, encrusting *Montipora* sp. and massive *Porites* sp. in this study showed recovery of color several months after the bleaching event (Fig. 1). Branched *S. pistillata*, however, was covered with algae and died the next year. Lower survival rates in branched corals compared to massive or encrusting corals have been reported previously in Mombasa and Okinawa following the extremely warm summer in 1998 El Nino [14, 60]. Given that branched corals have lower metabolic rates than massive corals, Gates and Edmunds [61] suggested that branched corals may have reduced capacity to respond to environmental changes. Loya *et al*. [14] also hypothesized that lower mass-transfer efficiency associated with branched morphology compared to encrusting and massive morphologies may be responsible for higher post-bleaching mortality in branched corals. Furthermore, lower tissue masses of branched corals may mean that branched corals have smaller energy reservoirs available for post-stress recovery [60]. This morphology-dependent survivorship may explain the variation in recovery among corals in this study. However, given that we did not measure tissue thickness or metabolic rates in our corals and that only three coral species were examined in this study, a definitive conclusion awaits further investigation.

### Bacterial community

Consistent with observations in other corals [26, 27, 62], *Gammaproteobacteria*, *Alphaproteobacteria*, and *Cyanobacteriia* dominated all coral samples collected in this study (Fig. 2). However, three coral species showed different microbiome dynamics following the bleaching event. For example, significant microbiome changes in *Montipora* sp. and *S. pistillata* were found in most pairwise comparisons throughout the sampling period, whereas microbiome changes in *Porites* sp. occurred primarily in comparisons across 2020 and 2021, but not within individual years (Fig. 4). Composition of microbiomes and their responses to environmental factors reportedly vary by coral species, sites, stress histories, and even between different compartments of a given coral colony [27, 28, 45, 63-65]. Our findings provide further evidence of the complexity of coral microbiomes. Unfortunately, in this study, microbiomes were not examined prior to the bleaching event. Therefore, whether microbiome changes in our corals, especially *Montipora* sp. and *Porites* sp., represent restoration of “pre-bleaching” bacterial communities cannot be definitively concluded. The lack of negative controls in this study and a relatively high number of PCR cycles (30 cycles) may also have resulted in some biases. Although we do not believe that these biases significantly affected the structure of dominant coral microbiomes, this possibility should be considered.

### *Endozoicomonas* abundance changes

Healthy corals host more abundant *Endozoicomonas* than bleached corals [27, 28, 30, 41, 66, 67]. Consistent with these observations, increases in *Endozoicomonas* relative abundance paralleled tissue color recovery in our corals, and a significant negative correlation between bleaching extent and *Endozoicomonas* abundance was found during recovery months (September 2020 to August 2021; Fig. 5D). However, the correlation was insignificant when data from the bleaching month were included (Fig. 5C), suggesting a more complicated association between coral bleaching and *Endozoicomonas* dynamics. In *A. millepora* in the Great Barrier Reef, Bourne *et al*. [30] identified shifts in coral microbiomes prior to visible signs of bleaching, including a decrease in *Endozoicomonas*. Although the low *Endozoicomonas* abundance in our *Porites* sp. in the bleaching month supports this hypothesis, both *Montipora* sp. and *S. pistillata* showed the highest *Endozoicomonas* relative abundances in the bleaching month. These findings suggest that *Endozoicomonas* decreases can also happen (or at least continue) after a bleaching event, raising the possibility that changes in *Endozoicomonas* abundance may not be an “early” indicator of coral stress in all coral taxa.

In an early study on Hawaiian corals, it was proposed that *Montipora verrucosa* possesses a lower lipid metabolic rate compared to *Porites compressa* [68]*.* In addition, branched corals have lower metabolic rates than corals of massive or encrusting morphotype [14, 61]. Although in this study we did not analyze lipid reserves, we assumed that *Porites* sp. in our study experienced earlier starvation due to faster consumption of its lipid reserve after bleaching, whereas *Montipora* sp. and *S. pistillata* showed delayed responses due to slower metabolism. According to this concept, *Endozoicomonas* abundance in a coral, instead of serving as an “early” stress indicator, more likely represents a “gauge” of coral health. This hypothesis helps to explain the correlation between *Endozoicomonas* deprivation and coral mortality in our *S. pistillata* and the decoupling of coral bleaching and *Endozoicomonas* abundance changes observed in other field surveys [28, 38] and laboratory experiments [43, 44]. Nevertheless, given that only three coral species were examined in this study and neither coral microbiomes before the bleaching event nor lipid reserves were available for our corals, further investigation is needed to test the global applicability and robustness of this hypothesis.

### *Endozoicomonas* composition shifts

Despite dynamics of *Endozoicomonas* abundance, taxonomic composition of dominant *Endozoicomonas* bacteria was largely stable in our corals throughout the sampling period, suggesting strong selection upon *Endozoicomonas* communities by coral hosts (Fig. 5B). However, most *Endozoicomonas* KTUs were common to multiple corals (Fig. 6). Although no negative controls were included in our microbiome sequencing, all our *Endozoicomonas* KTUs showed best matches to bacteria isolated/identified from seawater or marine invertebrates, indicating their presences in multiple corals were not likely due to contamination (Fig. 7). Furthermore, although most of our *Endozoicomonas* KTUs are likely novel *Endozoicomonas* species/strains (Fig. 7), two *Endozoicomonas* KTUs showed 100% sequencing identity to the *E. acroporae* strain Acr-14 and *E. atrinae* strain WP70, previously isolated from an *Acropora* coral and a comb pen shell, respectively [69, 70]. These findings suggest certain levels of flexibility in symbiosis between *Endozoicomonas* bacteria and corals and possibly also among dissimilar marine invertebrates. In addition, variations in levels of dominance among *Endozoicomonas* bacteria were detectable (Figs 5B and 6). For instance, KTU00005 was the dominant *Endozoicomonas* in *Montipora* sp. in the bleaching month, but it was surpassed by KTU00006 or KTU00001 during recovery. In *Porites* sp., the dominant KTU00001 showed a remarkable decrease in September 2020, when KTU00033 and KTU00048 became dominant. Species shuffling in the genus *Endozoicomonas* has been reported in *P. verrucosa* and *E. glabrescens* in response to DOC and dark treatments, respectively [43, 44] and in *Acropora muricata* during a reciprocal transplant experiment [45]. The present findings provide further evidence for *Endozoicomonas* shuffling in corals during recovery from a natural bleaching event. As *Endozoicomonas* bacteria show great genomic diversity [71], species shuffling in this bacterial genus may imply significant functional shifts in coral holobionts.

Interestingly, in our corals, we found a great variation in taxonomic composition of *Endozoicomonas* beside the dominant taxa (Fig. 6). Symbiont switching is defined as acquisition of novel symbionts from the environment, which was recently identified in algal symbionts of corals following natural disturbances [72, 73]. However, there is still no evidence of switching in coral-associated *Endozoicomonas*. Given that detection of rare bacteria can be strongly affected by sequencing depth, in our analysis, we filtered out low-abundance KTUs in an attempt to minimize this potential bias. Still, we found that several secondary *Endozoicomonas* appeared in recovery months in our corals, particularly in *Porites* sp., suggesting possible species switching in coral-associated *Endozoicomonas* community. Furthermore, most secondary *Endozoicomonas* KTUs were absent in seawater samples but were primary to other corals (Fig. 6), implying bacterial swapping among sympatric corals. Coral mucus harbors an abundant bacterial community [25, 74], in which dominance of *Endozoicomonas* is evident, as in *A. muricata* and *Porites astreoides* [27, 75]. Therefore, horizontal *Endozoicomonas* transfer between corals may be due to mucus secretion. Contamination with coral mucus may also explain the similarity of *Endozoicomonas* communities between the seawater sample in August 2021 and those in *Porites* sp. (Fig. 6). However, absence of certain *Endozoicomonas* bacteria in the bleaching month may also be due to the difficulty in detecting rare bacterial taxa. Accordingly, whether emergence of secondary *Endozoicomonas* in our corals is truly *de novo* requires further examination.

In this study, we analyzed bacterial community changes, particularly for *Endozoicomonas*, in three coral species following a bleaching event in 2020. These results challenge the early hypothesis that decreases of *Endozoicomonas* are linked to coral bleaching and suggest instead that *Endozoicomonas* abundance is likely correlated with host health. Furthermore, our findings provide evidence of species shuffling and possible switching within coral-associated *Endozoicomonas* following a disturbance. Flexibility in coral–*Endozoicomonas* symbiosis suggests the possibility of inoculating non-native coral hosts with *Endozoicomonas*. However, different dynamics of the same *Endozoicomonas* bacterium were observed between coral species, implying that effectiveness of *Endozoicomonas* as a coral probiotic may depend on coral species. Transient presence of secondary *Endozoicomonas* bacteria also suggests that repeat inoculation might be necessary for a long-term effect. Together, these findings show the potential of employing *Endozoicomonas* as a coral probiotic, which warrants further study.

## Supplementary Material

Supplementary_Table_S1_update2_ycae001

Supplementary_Table_S2_updated2_ycae001

Supplementary_Table_S3_updated2_ycae001

Supplementary_Table_S4_updated2_ycae001

Supplementary_Table_S5_updated2_ycae001

## Data Availability

Demultiplexed MiSeq data generated in this study are available on the NCBI Sequence Read Archive database under BioProject PRJNA1010003.
